# Performance of the HiberGene Group B Streptococcus kit, a loop-mediated isothermal amplification–based assay for GBS screening during pregnancy

**DOI:** 10.1007/s10096-022-04534-0

**Published:** 2022-11-30

**Authors:** Mireille Tittel-Elmer, Begoña Martinez de Tejada, Gesuele Renzi, Jacques Schrenzel

**Affiliations:** 1 Bacteriology Laboratory, Division of Laboratory Medicine, Department of Diagnostics, Geneva University Hospitals, Geneva, Switzerland; 2 Obstetrics Division, Departement of Pediatrics, Gynecology and Obstetrics, Geneva University Hospitals, Geneva, Switzerland; 3 Faculty of Medicine, University of Geneva, Geneva, Switzerland; 4 Genomic Research Laboratory, Division of Infectious Diseases, Department of Medicine, Geneva University Hospitals, Geneva, Switzerland

**Keywords:** Group B streptococci, LAMP assay, Intrapartum prophylaxis, Early onset neonatal sepsis, Screening, Pregnancy

## Abstract

Timely and accurate detection of Group B Streptococcus (GBS) carriage in pregnant women allows for targeted peripartum prophylaxis. Replacing culture-based screening by molecular biology assays enables faster results obtention, better targeted antibiotic prophylaxis, and reduces the laboratory workload. Here, we present a comparative analysis between a Loop Mediated Isothermal Amplification assay (HiberGene GBS kit) and culture (gold-standard). The HiberGene GBS kit showed a sensitivity of 97.9% and a specificity of 96.8% compared with culture. The limit of detection was estimated at 10^3^ cfu/ml and results were obtained within 30 min. HiberGene GBS assay can be used for peripartum GBS screening and targeted antibiotic prophylaxis provided sample processing can be swiftly performed around the clock.

## Introduction

Group B streptococci (GBS) belongs to the commensal vaginal and rectal flora and is present in 10 to 30% of women [1]. In pregnant women, this colonization is the primary risk factor for neonatal infections referred to as GBS early-onset disease (EOD). Prevention is based on intrapartum antibiotic prophylaxis (IAP) for GBS carriers, who are screened at 35–37 weeks of pregnancy using a culture-based approach. This strategy (screening and treatment) has been shown to efficiently reduce the incidence of EOD from 1.8 to 0.22 cases per 100 births [1]*.* Alternatively, a risk factor strategy, including previous delivery with invasive GBS disease, GBS bacteriuria during the current pregnancy, rupture of amniotic membranes for ≥ 18 h, birth at less than 37 weeks, and known GBS positive status during a previous pregnancy, can be used, at the expense of a lower efficacy [2]. Ideally, determination of maternal GBS carriage should be performed during the peripartum phase in order to permit the most adequate administration of the prophylactic treatment. Indeed, it has been shown that GBS carriage fluctuates during the pregnancy and more particularly from 35 to 37 weeks and the onset of labor. Reversion of 35–37 weeks GBS carriage occurs in about 10% of the cases and about 4% of negative GBS carriers are detected as positive at the onset of labor [3]. The use of molecular assays for GBS peripartum screening allows for targeting the correct population with analytical performances equivalent to culture [4].

Numerous commercially available PCR-based assays are now available including the Cepheid® Xpert® GBS that can be performed in the delivery room [4–7]. Here, we present a comparative analysis between the HiberGene Group B Streptococcus LAMP assay versus culture including a backup broth.

## Material and methods

Vagino-rectal swabs (ESwabs, Copan CE 0103, Brescia, Italy) were collected during the period of April 2021 to February 2022 in 153 patients and sent to the clinical bacteriology laboratory of Geneva University Hospitals. GBS detection by culture was performed on the WASPLab using CHROMID® Strepto B chromogenic agar plates (GBS CHROMID®, BioMérieux, Marcy l'Etoile, France) and a broth-enriched media (Copan, 476CE.A), which was systematically inoculated on another chromogenic agar after 24 h of growth. Suspect pink colonies were identified by matrix-assisted laser desorption ionization–time of flight mass spectrometry (MALDI-TOF/MS) (MBT Compass 4.1, Bruker Daltonics, Bremen, Germany) according to the manufacturer’s instructions.

As molecular-based assay, we used HiberGene HGGBSR2204, according to the manufacturer’s instructions. The HiberGene GBS kit is a LAMP-based assay performed in isothermal condition and thus made to be dispensed from a thermocycling unit, with the advantage of being more robust than PCR [8]. In brief, the vagino-rectal swabs used for the culture on agar plates were eluted in the appropriated buffer, and 80μL were lysed at room temperature for 20 min. The samples were denatured for 5 min at 105 °C and 25 μL added into each vial of the reaction strips prior to loading on the HiberGene GBS kit for GBS amplification. Control experiments using the Cepheid® Xpert® GBS were performed according to the manufacturer recommendation.

### Assessment of the limit of detection (LOD)

Real-time PCR positivity is based on fluorophore detection and expressed in cycling units, whereas for the LAMP assay is based on a more rapid target DNA amplification, shifting the turbidity of the amplification reaction and detected in minutes. To estimate the LOD of the HiberGene GBS assay, vagino-rectal ESwabs were spiked with GBS colony concentrations ranging from 3 × 10^2^ to 3 × 10^7^ cfu/ml. The time to positivity was plotted in minutes against the colony-forming unit/milliter (cfu/ml) as determined by vial cell counting on Columbia agar after overnight incubation. The LOD was derived from the lowest cfu/ml concentration correlating with a positive LAMP amplification.

## Results and discussion

Among the 153 specimens tested for GBS carriage, 48 (31%) were positive by culture. This rate does not reflect the real incidence, as we had selected 30 positive specimens for our analysis to test its clinical sensitivity. For this reason, the negative and positive predictive values cannot be calculated from our data. Among the 48 positive specimens, only 47 were taken in consideration due to one non-interpretable result of the Hibergene LAMP assay (Table 1, error). The sensitivity of the HiberGene GBS kit was 97.9% (95% [CI 88.9–99.6%]) corresponding to 46/47 positive specimens. The specificitey was 96.8% (95% CI [91.1–98.9%]) corresponding to 92/95 negative culture-based specimens (Table 1). The three false positive results were assessed using the Cepheid® Xpert® GBS kit, which returned negative and were therefore considered as “true” false-positive results. The performance of the Hibergene assay is therefore comparable with other commercialized GBS PCR assays, i.e. Cepheid® Xpert® GBS and GenomEra® GBS PCR, with the advantage of having a faster turnaround time than PCR assays [7, 9].

**Table 1:** Number of specimens according to the test results. Error: inconclusive result of the HiberGene GBS test due to technical error

HiberGene PCR	Culture		
	Positive	Negative	Total
Positive	46	3	49
Negative	1	92	93
Error	1	-	1
Total	48	95	153

**Fig. 1:**
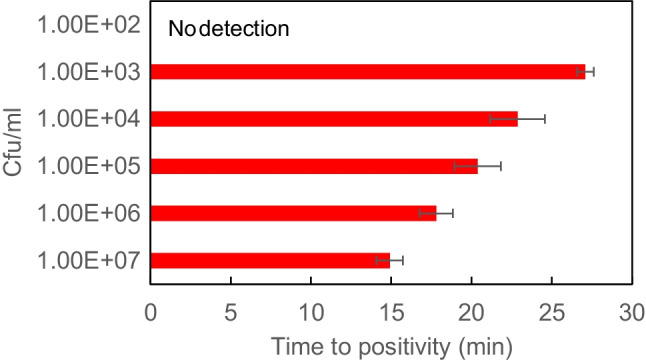
Negative clinical specimens were spiked with serial dilutions of GBS at concentrations ranging from 10^2^ to 10^7^ colony-forming units per ml (cfu/ml). The time to positivity measured by HiberGene GBS kit is expressed in minutes (min). Measurements were made in triplicate and errors expressed as SEM

Additionally, we found that the LAMP assay was able to detect specimens that revealed positive by enrichment broth, showing that the LAMP assay can accurately perform on low inocula. To evaluate the low inoculum detection by the LAMP assay and the issue of false positive detection, we investigated the LOD. The LOD corresponded to 10^3^ cfu/ml and a time of positivity of 27.1 ± 0.5 min (Fig. 1). Occasionally, specimens with 10^2^ cfu/ml returned positive but duplicates provided unreproducible data (data not shown). The serial dilutions spanning from 10^3^ to 10^7^ cfu/ml showed a consistent and linear decrease in time to positivity (Fig. 1). These data demonstrated that the LOD of the HiberGene GBS kit was equivalent to other commercially available molecular-based assays [9].

The HiberGene GBS assay is an accurate and rapid test, requiring less laboratory skills and time than culture. Compared to existing PCR assays, it has the advantage of a lower price but needs to be executed by laboratory technologists. This implies having a diagnostic laboratory operating on a 24 hours basis to benefit from this rapid turn-around time. GBS carriage results should be available to allow targeted intra-partum prophylaxis and for at least 4 hours. Cepheid® Xpert® has been shown to allow for correct prophylaxis in the same number of women than antenatal culture but targeting the highest risk population [4]. As the HiberGene GBS assay performs faster or at least as fast as the Cepheid® Xpert®, this suggests that the HiberGene GBS can be utilised to rapidly and accurately detect GBS carriage in pregnant women provided laboratory staff is available at the vicinity and analyzing around the clock.
